# Supplementary figures and images for: Pilot study evaluating everolimus molecular mechanisms in tuberous sclerosis complex and focal cortical dysplasia
Source: PLoS One. 2022 May 19;17(5):e0268597. doi: 10.1371/journal.pone.0268597 (PMC9119437; doi:10.1371/journal.pone.0268597)

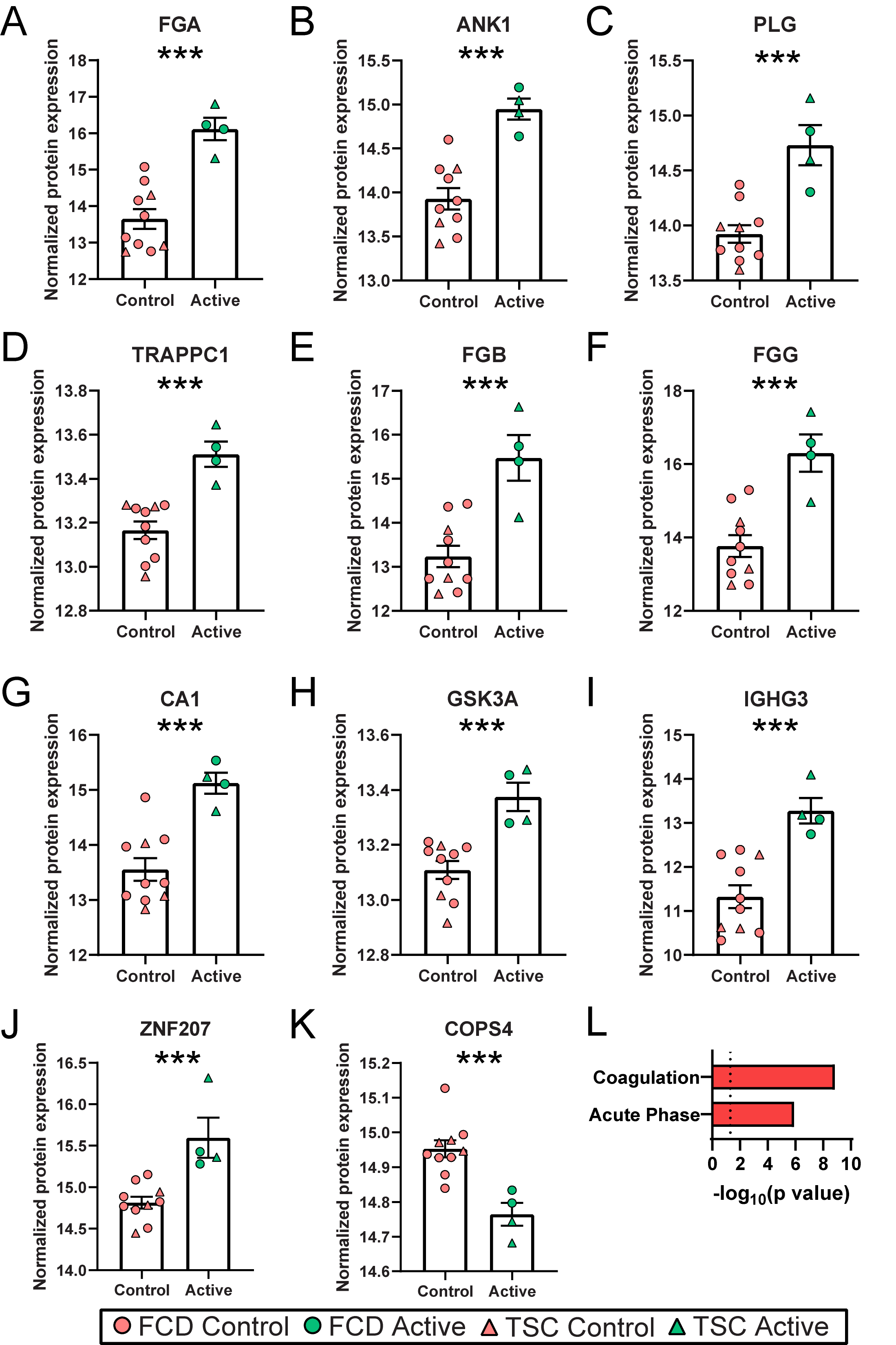

Supplement: S1 Fig — A-K) Expression of the 11 altered brain proteins at FDR<15% in Active versus Control participants, in order by increasing p value. Distribution indicates that these differences are not all due to Active TSC participants, in which there was segregation of TSC cases on the PCA in Fig 3A. Error bars indicate SEM. L) The 11 altered proteins were associated with 2 signaling pathways having a p value of overlap < 0.05 and |z| > 2. The same four proteins (FGA, FGB, FGG, PLG) were associated with activation of the coagulation system (p value of overlap = 1.45 x 10^−9^, z = 2.00) and acute phase response signaling (p value of overlap = 1.23 x 10^−6^, z = 2.00). (TIF) [file pone.0268597.s008.tif]

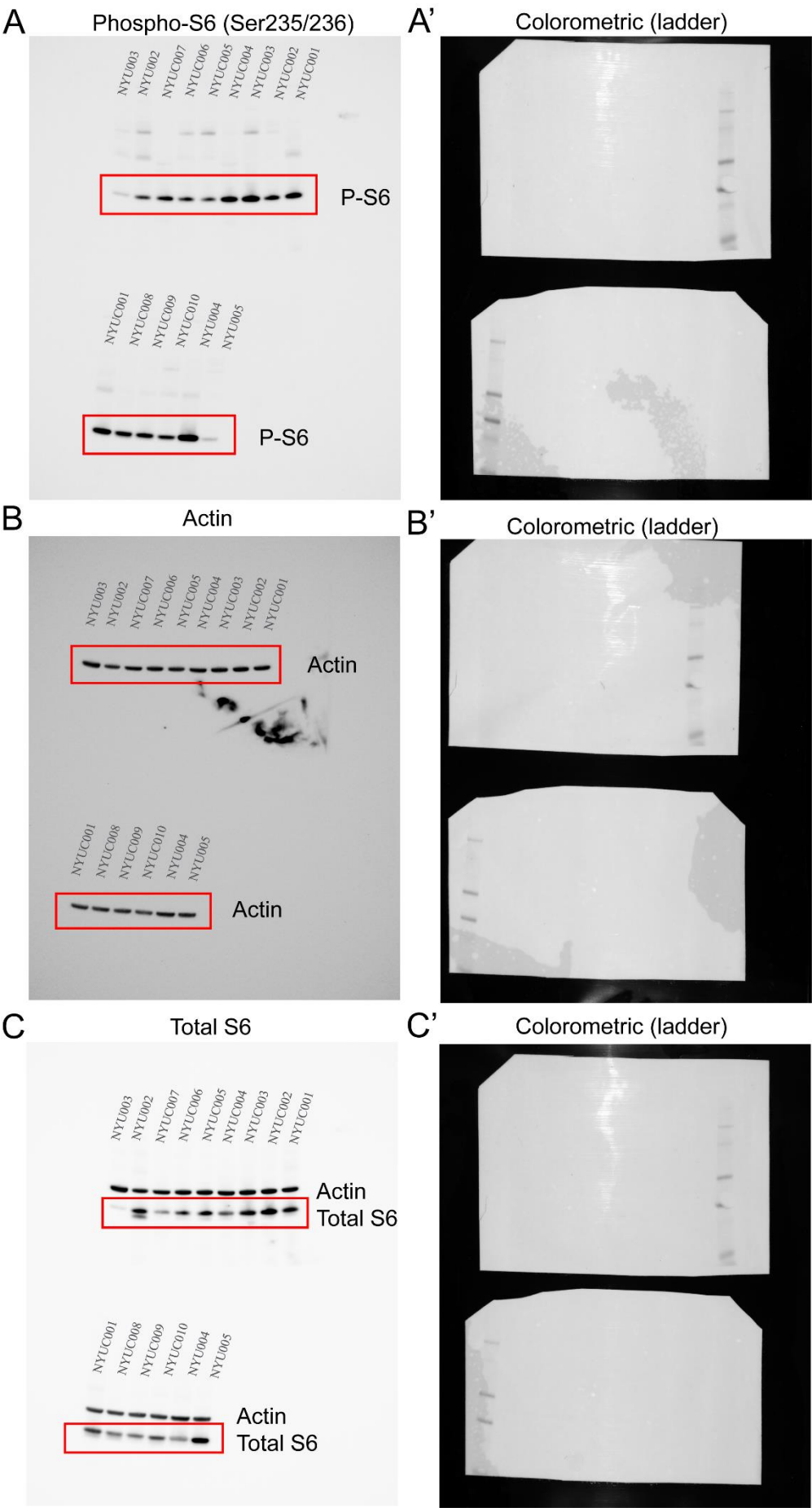

Supplement: S1 Raw image — A) All cases (n = 14) were evaluated by western blot for phospho-S6 (Ser235/236) quantification on 2 blots, as depicted in Fig 1A. Bands were visualized after ECL on a BioRad ChemiDoc. Quantification was performed on bands in the red outlined box in Fiji ImageJ. One sample (NYUC001) was included on both gels to allow for normalization across blots for all samples. A’) Corresponding ladder for panel A is shown in the colorometric brightfield image. B) On the same blots, actin was evaluated after stripping the phospho-S6 (Ser235/236). B’) Corresponding ladder for panel B is shown in the colorometric brightfield image. C) On the same blots, total S6 was evaluated with no stripping (both actin and total S6 are present). C’) Corresponding ladder for panel C is shown in the colorometric brightfield image. (PDF) [file pone.0268597.s009.pdf]

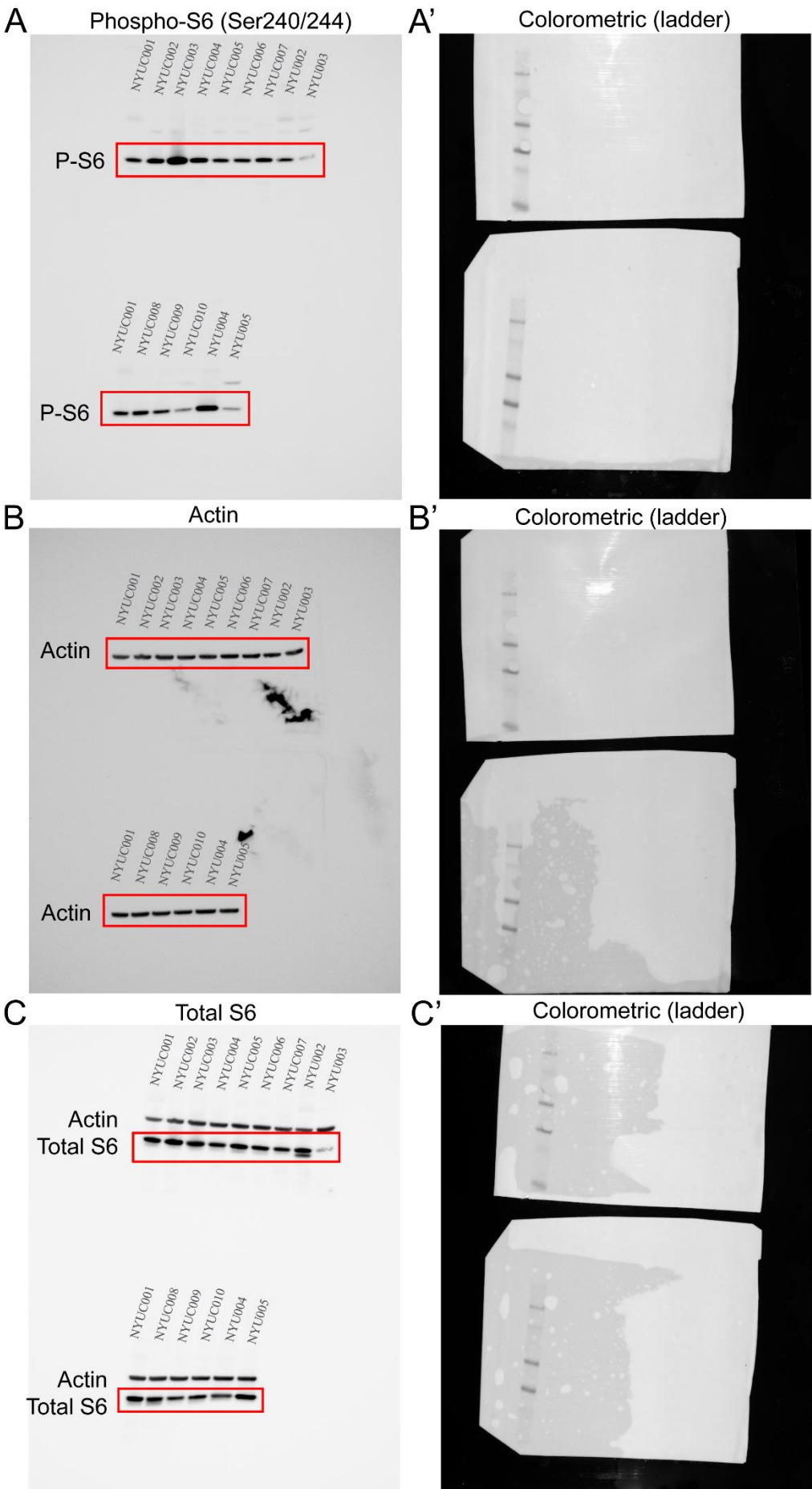

Supplement: S2 Raw image — A) All cases (n = 14) were evaluated by western blot for phospho-S6 (Ser240/244) quantification on 2 blots, as depicted in Fig 1A. Bands were visualized after ECL on a BioRad ChemiDoc. Quantification was performed on bands in the red outlined box in Fiji ImageJ. One sample (NYUC001) was included on both gels to allow for normalization across blots for all samples. A’) Corresponding ladder for panel A is shown in the colorometric brightfield image. B) On the same blots, actin was evaluated after stripping the phospho-S6 (Ser240/244). B’) Corresponding ladder for panel B is shown in the colorometric brightfield image. C) On the same blots, total S6 was evaluated with no stripping (both actin and total S6 are present). C’) Corresponding ladder for panel C is shown in the colorometric brightfield image. (PDF) [file pone.0268597.s010.pdf]
